# Perspectives on AI-Driven Nursing Science Among Nursing Professionals from China: A Qualitative Study

**DOI:** 10.3390/nursrep15060218

**Published:** 2025-06-14

**Authors:** Yi Chen, Fulei Wu, Wen Zhang, Weijie Xing, Zheng Zhu, Qingmei Huang, Changrong Yuan

**Affiliations:** School of Nursing, Fudan University, Shanghai 200032, China; chenyiabc@163.com (Y.C.); wufulei@fudan.edu.cn (F.W.); zhangwennurs@fudan.edu.cn (W.Z.); xingweijie@fudan.edu.cn (W.X.); zhengzhu@fudan.edu.cn (Z.Z.)

**Keywords:** nursing, artificial intelligence, scientific research, nursing education, clinical practice, qualitative study

## Abstract

**Background**: As artificial intelligence (AI) continues to advance in healthcare, limited research has explored how nursing professionals perceive its integration into clinical practice and education—particularly among those directly involved in AI-driven initiatives. This qualitative study aimed to investigate the perceptions, experiences, and expectations of nursing educators and clinical practitioners regarding the application of AI in nursing and to provide insights for the advancement of AI-driven nursing science. **Methods**: A descriptive qualitative design was employed. Between September and December 2024, semi-structured interviews were conducted with 12 nursing professionals from universities and hospitals in Shanghai, Suzhou, and Chengdu, China. Participants were selected using maximum variation sampling, and data were analyzed using content analysis. **Results**: Three major themes and eleven sub-themes were identified: (1) The potential of multi-perspective development of AI-driven nursing science and practice, including aiding in decision-making, assisting with writing nursing documents, helping in care practices with high exposure risks and heavy physical exertion, and supporting the development of nursing activities. (2) A multi-dimensional response to the wave of intelligent nursing research and practice: education and scientific research come first, then we fully explore the application scenarios, and then conduct deep interdisciplinary integration. (3) Obstacles for intelligent nursing research and practice: interaction factors of “human–technology–machine” for application, transformation, and promotion; financial support and continuous investment; the controversy behind the intelligent maturity level; and application risk and fault tolerance. **Conclusions**: Participants emphasized the importance of evidence-based, cautious, and context-sensitive application of AI technologies to ensure that intelligent nursing evolves in alignment with clinical realities. The findings suggest a need for strengthened policy, education, and resource allocation to support the sustainable integration of AI in nursing.

## 1. Introduction

Artificial intelligence (AI) was first introduced at the Dartmouth Conference in 1956 [1], aiming to simulate and enhance human intelligence through theories, methods, and technologies encompassing problem-solving, pattern recognition, and decision-making. As a core component of the field of intelligence science, AI spans robotics, speech and image recognition, natural language processing, expert systems, machine learning, and computer vision [2]. Its rapid development is profoundly transforming various industries, with significant potential to improve healthcare practices [3].

The integration of AI into scientific research is reshaping methodologies and practices; researchers need to adapt to and leverage these advancements [4]. Understanding nursing professionals’ perspectives on AI-driven nursing practices, research, and education is essential for facilitating its meaningful integration into the field [5,6]. However, there is a lack of qualitative research exploring the views of nursing professionals who have directly participated in AI-driven projects. This study aims to fill this gap by exploring their perceptions and experiences, providing a comprehensive understanding of AI’s role in nursing and offering practical recommendations for its future implementation.

Over the past decade, research on technologies based on AI in healthcare has increased, demonstrating significant potential to support and enhance patient care. However, a scoping literature review showed that most studies explored the AI technology development and formation (testing) phases, followed by the implementation and operational phases. One-third of papers (33.3%) were reported without the involvement of nurses [7]. As AI tools and systems become more prevalent in clinical settings, capturing the perspectives of nurses who engage with these technologies is crucial.

Recent quantitative studies have increasingly explored nurses’ attitudes, levels of preparedness, and perceived barriers related to the integration of AI in healthcare. Several studies have focused on nursing students’ awareness and readiness for AI. For example, one study investigated undergraduate nursing students’ awareness of the role of AI in healthcare [8], while another examined the relationship between AI readiness and individual innovativeness among nursing students [9]. Similar research from Palestine assessed nursing students’ attitudes toward the application of AI [10]. In clinical practice, nurses’ knowledge, attitudes, practices, and perceived barriers regarding AI have also been studied, with some findings suggesting these factors can predict nurses’ intention to stay in their nursing jobs [11]. A recent study found that over half of the surveyed registered nurses had used or were familiar with ChatGPT and held generally positive attitudes toward its use, although perceived risks influenced their willingness to adopt it in practice [12]. In addition, Alruwaili et al. evaluated nurses’ awareness and attitudes toward AI tools in clinical settings [13].

Existing qualitative studies have provided deeper insights into the facilitators and barriers to AI integration from various nursing perspectives. Some studies have explored these issues across different professional roles [6], while others have examined experiences with generative AI in educational settings [14]. For instance, Badawy and Shaban conducted a qualitative study to understand geriatric nurses’ perspectives on adopting AI in elderly care [15]. Nursing educators’ perspectives on the integration of AI into academic settings were also examined in another qualitative investigation [16]. Other studies have focused on regional contexts. A qualitative study in Jordan explored nurses’ views on the application of AI in nursing practices [17]. In Turkey, physicians, nurses, and patients were surveyed regarding their perceptions of AI and robotic nurses, including their attitudes toward integrating these technologies into healthcare delivery [18]. Furthermore, Alkan et al. conducted a phenomenological study to investigate whether nurses perceive AI as a threat or an opportunity in patient care. Most participants believed that, when guided by clear regulations and appropriate oversight, AI could effectively support, enhance, and improve the quality of nursing care [19].

However, there is still a lack of qualitative research that explores the perspectives of nursing professionals who have been directly involved in the design and implementation of AI-driven initiatives. Existing studies have predominantly focused on general perceptions of AI in healthcare or the views of technical stakeholders, without capturing the lived experiences and professional insights of nurses who are actively engaged in these transformative projects.

To address this gap, this study aims to explore the perspectives of nursing professionals who have participated in the design and implementation of AI-enabled nursing interventions. By doing so, we seek to provide a comprehensive understanding of the opportunities and challenges associated with AI integration in nursing practice and to offer practical recommendations for its future development and application.

## 2. Materials and Methods

### 2.1. COREQ Checklist

This study was conducted using a qualitative methodology [20] and thematic analysis [21]. In-depth interviews with semi-structured questions were used to guide the interviews. The reporting of this study complies with the consolidated criteria for reporting qualitative research (COREQ) recommendations [22].

### 2.2. Participants and Recruitment

A purposive sampling method and maximum variation sampling method were adopted to ensure a diverse range of valuable insights on the research topic. In the new course AI for Nursing Science, which began in September, 2025 at the authors’ university, and at the conference of the Nursing Branch of China Health Information and Health Medical Big Data Society, the first author came into contact with several hospital-based and academic instructors who had experience with AI integration in clinical practice or research. With the referral from one participant (N2), the first author established contact with two additional clinical instructors (N10 and N12) who had prior experience implementing AI integration projects in their nursing practice.

Nursing professionals were recruited from three distinct medical universities and three hospitals. Of the 13 initially identified candidates, 1 declined participation due to scheduling conflicts. The remaining 12 interviewees successfully completed the interviews.

### 2.3. Inclusion and Exclusion Criteria

Inclusion criteria: (a) registered nurses or nursing educators; (b) obtained at least a master’s degree and have at least 3 years of rich professional experience; (c) willing to actively participate in in-depth conversations; (d) have experience in applying AI technology to empower nursing research or practice. Exclusion criteria: (a) language or communication barriers; (b) unable to guarantee sufficient participation time; (c) have conflicts of interest or biases; (d) suffer from serious psychological or other health problems.

### 2.4. Qualifications of the Research Team

The first author is a doctoral student. The other authors are PhD-level teachers. The authors are working at the university, which is part of the C9 League (an alliance of top universities) in China. The authors who participated in this study are familiar and experienced with qualitative research methods. All of the interviews were conducted by the first author for neutrality in the interview process. The interviews were transcribed by two research assistants.

### 2.5. Data Collection

This study employed a descriptive qualitative research approach. Following a literature review and consultation with nursing informatics experts, an initial interview guide was developed. A pilot interview was conducted with a faculty member teaching the Nursing Information and Big Data course, after which the guiding questions were refined to form the final interview outline. The interviews focused on four key areas: (1) perceptions of the current development of AI-driven nursing science, (2) the impact of AI on the nursing discipline, (3) strategies for integrating AI into intelligent nursing practices, and (4) challenges encountered in AI-related project implementation. The complete interview guide is provided in Appendix A.

From September to December 2024, ten interviews were conducted face-to-face in a quiet conference room, while two were held via Tencent Conference with the camera turned on, a widely used online meeting platform in China. Each interview lasted between 33 and 59 min. The interviews were conducted by the first author, a female Master’s researcher in nursing science with qualitative research training, prior experience in qualitative studies, five years of clinical nursing experience, and two years in nursing education. A certain level of familiarity had been established between the interviewer and the participants prior to the interviews. This familiarity, developed through previous professional or academic interactions, was intended to build trust and facilitate open, in-depth conversations.

Before each interview, participants were informed of the study’s purpose, and their confidentiality and privacy were assured. Upon obtaining consent, audio recordings were made. All interviews were conducted one-on-one in a private and quiet office setting with no other individuals present to ensure confidentiality and minimize distractions. During the interviews, non-verbal cues such as body language and facial expressions were observed, and clarifications or follow-up questions were asked as needed. The sample size was determined based on data saturation, whereby if no new themes emerged after interviewing two additional nursing professionals, the sample size was considered adequate. No follow-up interviews were conducted. Each participant was interviewed once.

Within 24 h of each interview, two researchers independently transcribed the recordings into textual data. All interviews were conducted in Mandarin Chinese, and the transcripts were also transcribed in the same language. Transcripts were then returned to participants for verification. Relevant excerpts used in the manuscript were subsequently translated into English for reporting purposes. Microsoft Word and NVivo 14.0 were used for data management and analysis.

### 2.6. Data Analysis

Two nursing doctoral students analyzed the transcribed data (in Chinese) using content analysis. The specific steps were as follows: (1) transcribe the interview audio recordings into text word by word; (2) identify meaningful sentences or paragraphs through reading and use them as units of analysis; (3) assign codes reflecting the essential meaning according to the units of analysis to generate codes; (4) analyze and compare the codes with similar meanings, summarize categories, and verify them; (5) analyze the categories, extract themes, determine the interrelationships, and construct a conceptual form; and (6) return to the original data to verify whether the theme is truly suitable for the original data and make necessary revisions. For publication purposes, selected quotations were translated into English by bilingual researchers with expertise in qualitative research and nursing research. To ensure accuracy and conceptual equivalence, a back-translation process was employed, whereby a second bilingual researcher independently translated the English excerpts back into Chinese. Discrepancies were discussed and resolved by the research team to maintain fidelity to the original meaning. To ensure that the findings reflected participants’ perspectives rather than researchers’ preconceptions, we employed strategies such as reflexive journaling and team-based coding discussions to minimize potential bias and enhance analytical neutrality.

### 2.7. Ethical Considerations

This study was approved by the Ethical Committee of School of nursing in Fudan University (Approval IRB # 2024-6-4). Prior to the interviews, all participants were thoroughly informed about the purpose of the study and provided verbal consent. To ensure confidentiality, all interview-related data, such as descriptions and recordings, were stored on a password-protected computer, with access strictly limited to the authors. Backup files were securely stowed away in locked file cabinets. Moreover, each participant was assigned a unique identification code to anonymize their responses. All personal identifiers were carefully anonymized in transcripts and associated documents to ensure complete dissociation between the data and individual identities.

### 2.8. Rigor and Reflexivity

Purposive sampling recruited nursing professionals of different ages and genders, all experienced in AI-driven nursing projects, ensuring representativeness. Open-ended questions and active listening encouraged authentic responses.

Participants from hospitals and universities represented diverse nursing roles, enhancing transferability.

To ensure methodological rigor, researchers documented key details, including interview time, location, and participant demographics, while maintaining a comprehensive research log. Multi-person coding with cross-checking ensured accuracy and consistency.

All original data, including interview recordings and transcripts, were preserved, with a clear audit trail to enhance transparency and reliability.

## 3. Results

### 3.1. Study Participants

A total of 12 interviewees were finally included. Participants were from Shanghai, Suzhou, and Chengdu across three cities. The participants had a mean age of 38.83 years (SD = 7.71; range: 31–60), the average number of years of participation in AI-related fields was 6.17 years (SD = 4.65; range: 3–20), and the average teaching experience in the nursing field was 10.17 years (SD = 7.85; range: 3–30). Characteristics of the participants are shown in Table 1.

### 3.2. Major Themes

The codes and themes from the analysis were organized (Table 2), and we extracted three major themes and 11 sub-themes: (a) The potential of multi-perspective development of artificial intelligence-driven nursing science and practice: aiding in decision-making; assisting with writing nursing documents; helping in care practices with high exposure risks and heavy physical exertion; supporting the development of nursing activities. (b) A multi-dimensional response to the wave of intelligent nursing research and practice: education and scientific research come first, then we fully explore the application scenarios, and then conduct a deep interdisciplinary integration. (c) Obstacles to intelligent nursing research and practice: interaction factors of “human–technology–machine” for application, transformation, and promotion; financial support and continuous investment; the controversy behind the intelligent maturity level; and application risk and fault tolerance.

### 3.3. Subtheme 1: The Potential of Multi-Perspective Development of Artificial-Intelligence-Driven Nursing Science and Practice

Artificial intelligence is based on the collaborative development of data science. From the perspective of clinical nursing practice, it has rich multi-dimensional and multi-modal interactive data with doctors, healthcare support staff, patients, and family caregivers throughout nursing assessment, nursing diagnosis, nursing intervention, and nursing evaluation. This has given rise to the application potential of using AI to assist decision-making, assisting with in writing nursing documents, help in care practices with high exposure risks and heavy physical exertion, and support the development of nursing activities.

#### 3.3.1. Aid in Decision-Making

In the field of nursing, emerging research related to AI is focused on the establishment of prediction models and clinical nursing decision systems supported by data and various algorithms.

*‘To the best of my knowledge, making recommendations, making predictive models, making knowledge graphs, they are hot topics for us right now.’*

*(N7)*

*‘For example, the clinical nursing decision system, it may give greater support to younger nurses. Experienced nurses are adept at discerning specific signals; when these indicators emerge, they can ascertain that a patient may be at an elevated risk for a particular condition, while it is hard to judge the circumstances for young nurses. Luckily, if AI-based nursing decision support system comes out, it may give hints to the unexperienced nurses. By enabling younger nurses to identify potential patient risks at an earlier stage and in a timely manner, we enhance the equity of healthcare provision.’*

*(N11)*

However, due to the limited quantity and quality of data, there are few well-trained models to assist in decision-making.

*‘It (AI-driven nursing research and practice) must start at the research level, but currently, there are very few well-trained predictive models in the field of medicine and health.’*

*(N6)*

#### 3.3.2. Assisting with Writing Nursing Documents

Due to the prominent language performance capabilities of generative AI, the vast amount of nursing records and documents in the nursing field serve as the entry point for the integration of AI with nursing.

*‘In terms of medical/nursing documents, large language models can help you write them well and reduce the burden on nursing staff… (You know, a substantial amount of paperwork is always waiting for the nurses)’*

*(N1)*

In nursing education, just like in other fields, it can assist with writing related documents.

*‘Since the emergence of Chat-GPT, we have also used some text generation tools in our own work. For example, when we are not very familiar with a certain field, we can ask the model for some rough ideas as a framework for sorting out.’*

*(N2)*

*‘Perhaps it can help nurses write a short essay, which can help them do better in health communication.’*

*(N8)*

#### 3.3.3. Help in Care Practices with High Exposure Risks and Heavy Physical Exertion

The advantages of AI lie in its intelligence and automation. Nursing stands out among medical professions for bearing the heaviest workload within hospital settings, encompassing a multitude of repetitive, hazardous, and physically strenuous duties. Therefore, under scientific design, it is necessary to achieve intelligence and automation in the field of nursing.

*‘Large language models are designed for generating responses. It is poised to become a promising and flourishing trend within the nursing profession. Its advantage is that it won’t get tired… I think if a nurse in the outpatient department has to answer the same question 100 times a day, it’s quite hard for her/him. (Laughs)’*

*(N11)*

*‘Nowadays, the majority of patients require assistance to turn over in bed, which is very physically demanding. So, these robots are actually very valuable and meaningful… I think robots can help us, and also take over some risky tasks, such as preparing chemotherapy drugs, or handling radioactive or other occupational exposure tasks, like suctioning for infectious disease patients. I think these can also be done by robots for occupational protection.’*

*(N7)*

*‘In addition, there are some high-risk operations. For instance, nurses are required to handle radioactive materials, such as those involved in X-ray procedures. If there is a robot, I can let it operate inside, which can avoid people being exposed to radiation. Moreover, in the operating room, if we can use robots to move patients, many patients need to be transferred from the bed to the trolley after the operation, and this process can also be done by robots. Of course, after many years of development, robots may be fully capable of handling high-intelligence work, but at this stage, I think we can start with repetitive, physically demanding and dangerous tasks, and let robots take over these tasks for us.’*

*(N12)*

Using intelligent robots to replace some of the work of nursing staff can actually give them more time to provide necessary humanistic care to patients and carry out other more creative and complex tasks.

*‘Indeed, the integration of AI is anticipated to alleviate the burden of repetitive tasks on medical staff, enhance the treatment experience for patients, and potentially afford nurses additional time to dedicate to the nuanced care of patients—care that machines are incapable of providing.’*

*(N11)*

*‘Nowadays, even the service provided by humans to patients is standardized. Now we require (nursing staff to) show care, but under our heavy workload, how can we show care? (Waves right hand excitedly) Can you take care of their emotions? It’s good if you can finish the work. (Pause) The so-called highest ideal is that if a lot of basic work is replaced by machines, you may have time to care for patients (laughs), right?’*

*(N1)*

*‘AI may replace some of our repetitive labor. This actually puts higher demands on our nursing staff. You may need to spend more time dealing with more complex tasks that require judgment, problem-solving, and creative thinking, critical thinking, etc., to discover complex conditions and make pre-judgments. In fact, this requires higher thinking skills from our nurses.’*

*(N2)*

#### 3.3.4. Support the Development of Nursing Activities

The activities in the field of nursing cover various levels of assessment, monitoring, care behaviors, education, follow-up, and other tasks. Based on different types of AI, AI also has strong advantages in assisting with various nursing activities.

*‘Although there are currently guidance and consultation robots, they are in the form of text. Could they be developed into voice-based ones? Because many patients, especially the elderly, although they have registered successfully, it is their younger relatives who do it for them. In many cases, the patients are not present when filling out some information for pre-consultation and assessment, and the information may not be accurate. So, from the perspective of computer usage preferences, voice can be used to connect with patients and conduct innovative assessments and pre-consultations through voice interaction.’*

*(N8)*

The participants held views that utilizing embodied intelligence for nursing care and education work is essential nowadays or a few years down the line.

*‘Regarding embodied intelligence, if we develop a good nursing model, it can be used in both homes and nursing homes, which are both in need. Compared to regular nurses in hospitals, caregivers have much poorer care skills. Moreover, the daily care procedures in hospital wards are very standardized and strict. Therefore, whether in nursing homes or in our wards, care is a scarce resource… Many elderly people do not lack housing but have no one to take care of them at home, which is a significant social issue in elderly care. If we can convert professional nursing experience into such robots, automatic care robots, there would be a huge demand for them.’*

*(N1)*

*‘During your study at the college, could you build a virtual simulation environment to train robot nurses, just like training regular nurses, where they have to attend classes, perform operations, and take exams. Is it feasible to establish such a system? Indeed, it warrants consideration.’*

*(N1)*

### 3.4. Subtheme 2: Multi-Dimensional Response to the Wave of Intelligent Nursing Research and Practice

#### 3.4.1. Education and Scientific Research Come First

“Science and technology are the primary productive force”. In the view of the interviewees, against the backdrop of the rapid development of AI, education and scientific research are one of the most important ways to gradually transition the nursing field to intelligent nursing research and practice.

*‘To promote the use of AI in the nursing field, there must be top-down design, which requires some groundwork. For example, the most important groundwork is the cultivation of talents.’*

*(N10)*

Students ought to embrace a more proactive approach to their education, aligning their active learning strategies with the evolving demands of the era.

*‘As society marches forward, many AI products, as well as some services, brands, and concepts are constantly emerging and infiltrating. Isn’t it a way of embracing AI when we take the initiative to understand and use them?’*

*(N1)*

*‘Currently, AI-driven nursing science remains research-focused, with limited clinical application. Widespread adoption has yet to occur, highlighting the need for self-directed learning through literature review, which could significantly advance the field.’*

*(N4)*

The content of active learning is rich and diverse, such as the classification of the AI system, the basic principles of AI technology, and the background knowledge of the nursing profession.

*‘One has to know the so-called programming logic. Now, things like Chat-GPT can help you with programming. You need to know the overall programming logic and how to implement it, right? If you can’t even figure out the data type and don’t know the basics, it would be very troublesome… Recently, I’ve gradually realized that the professional background is very important. Otherwise, the results will be rather strange.’*

*(N5)*

In addition, when educators carry out teaching activities, they should also guide students to open up their minds as much as possible.

*‘We need to open up our minds, keep up with the times to learn these AI technologies, and at the same time, I think we should also cultivate our critical thinking ability, as well as the ability to raise and solve problems. We should develop more in this regard.’*

*(N2)*

*‘I think scientific research sometimes requires wild imagination… In fact, applying AI to our clinical practice, on the one hand, requires resources, and on the other hand, requires the courage to think, and the spirit of daring to think.’*

*(N10)*

*‘I think the course our team has developed (AI-driven Nursing Research and Practice) is actually to broaden everyone’s horizons and provide ideas. I think it is very important. From a cutting-edge perspective, we need to let our students know in which aspects AI can be combined with their future work in their field, and what can be done in this direction.’*

*(N3)*

In the interaction between educators and students, students’ unique ideas and inspirations will better connect the application of AI to nursing scenarios and also promote the development of educators’ ability to apply AI to empower nursing research and practice, that is, teaching benefits teachers as well as students.

*‘In fact, students are very creative, especially at the postgraduate stage, which is quite different from the undergraduate stage. Education is not merely a one-way transmission of knowledge but an interactive process. Take our small projects on generative AI as an example. In fact, I have learned a great deal from my students. Due to their intense focus on this issue, they have immersed themselves in extensive reading. Then they explained to me what exactly is going on, including the relationship between generative AI, large language models, and as well as different model adaptation methods and their respective advantages and limitations. This reciprocal learning process highlights the importance of focusing on the practical application of AI, where both students and educators expand their knowledge and skills, ultimately benefiting the field.’*

*(N4)*

#### 3.4.2. Fully Explore the Application Scenarios

“Good steel should be used on the blade’s edge”, and the use of AI in the field of nursing must also find the right application scenario.

*‘Students should contemplate the specific scenarios where AI can be integrated into future nursing clinical practice. These scenarios are dynamic and may evolve into specialized niches within nursing. Delving deep into these scenarios fosters scientific thinking and questioning skills, bridging nursing research and education.’*

*(N2)*

*‘Although robots are not yet widely used in nursing, their adoption in other service industries provides valuable insights. Exploring potential applications in nursing requires a proactive approach—identifying relevant technologies, testing available platforms, and initiating pilot projects. If you’re not even inclined to attempt, merely waiting for others to deliver solutions to you, is that truly sufficient?-one must be willing to experiment.’*

*(N1)*

#### 3.4.3. Deeply Interdisciplinary Integration

To achieve AI- driven care models, products, and robots, it is necessary to set common goals based on real needs and carry out interdisciplinary integration.

*‘No matter which discipline, in fact, the country has always advocated the cross-disciplinary, right? For engineering personnels, only with a foundational understanding of nursing can its computational research and applications truly empower and enhance the field, like adding wings to a tiger… To be thoroughly integrated, there will be output, otherwise I think it is quite difficult.’*

*(N10)*

*‘We often depend on innovative ideas, yet the realization of such concepts in AI necessitates the collaboration of a multidisciplinary team, leveraging their collective expertise to bring these ideas to fruition.’*

*(N9)*

The needs presented by the caregiver should be based on real-world urgent needs, not “pseudo needs” (N5).

*‘Our efforts invariably culminate in serving the clinical settings, as hospitals prioritize practicality: how to reduce the work burden of nurses, how to enhance patient access to nursing services, and ultimately enhancing patient outcomes? In the end, it comes down to practical concerns…’*

*(N7)*

Based on common goals and ideas, multiple collaborations lead to deep cross-pollination and high-quality output.

*‘My collaboration with the School of Computer Science extends beyond a one-off partnership; it is imperative that we share a unified vision and practical objectives with them, as they are in the midst of transformation, they have technology to achieve, but there is no application scenario. They really need us… Indeed, fostering scientific collaboration at the level of camaraderie can be highly beneficial.’*

*(N5)*

*‘Now since that time (with the school of Computer Science to cooperate on a project), we often have further communication, and some related topics, we also encourage students to apply for student innovation and entrepreneurship competitions at college level, and we are now working on related things together.’*

*(N12)*

The establishment of a stable multidisciplinary team is important for long-term sustainable development and integration of technologies.

*‘During my studies in Canada, the hospital featured a specialized research center. Principal Investigators (PIs) led healthcare teams, tackling diverse issues while emphasizing nursing informatics. The center housed IT experts focused on supporting the hospital’s research teams, fostering high-efficiency communication and collaborative technology development with the information center.’*

*(N3)*

*‘During my visit to a nursing research institute, I saw a mix of engineers, biology students, and materials scientists aiding nursing staff. Here, nursing students gain diverse insights during experiments—materials scientists and AI experts offer differing approaches. Gradually, we learn to absorb engineering thoughts or learn more about the AI logics or whatever… I think.’*

*(N10)*

### 3.5. Subtheme 3: Obstacles to Intelligent Nursing Research and Practice

*In the era of rapid development of AI, there have been various kinds of researches on intelligent nursing research and practice, but meanwhile, obstacles follow up. As N6 said, “Despite the publication of countless studies, only few ultimately benefit patients and achieve clinical translation.”*

#### 3.5.1. Interaction Factors of “Human–Technology–Machine” for Application, Transformation, and Promotion

##### Insufficient Mutual Understanding Between Different Individuals

Human willingness is an important factor in the application, transformation, and promotion of AI in the field of nursing, including the understanding and willingness of leaders and the research willingness of researchers.

*‘I think the biggest challenge is people, whether people are willing, willing to use, willing to push for some change in this thing.’*

*(N1)*

Experts raised questions about implementing AI-driven nursing research, such as ‘the proportion of nursing components in the process of multi-disciplinary cooperation’? Nowadays, in the process of developing intelligent nursing research and practice, we also need to obtain more tolerance and understanding from peer experts.

*‘This year, during the student research proposal defense, whose proposal was related to the large language model, experts asked many questions to raise doubts…’*

*(N5)*

*‘Perhaps the key question is: to what extent must nursing be integrated into our multidisciplinary collaboration for it to qualify as a nursing project? When my students draft their proposals, there’s a common perception that the core technical aspects are outsourced, leaving the distinct nursing contribution unclear. In reality, I believe we need a more inclusive and flexible research environment to foster innovation among our graduate students.’*

*(N4)*

The relationship between mutual understanding and achievement between teachers and students is also the focus of attention in the application, transformation, and promotion of AI in the field of nursing.

*‘I think we hope that we can provide some more inclusive and relaxed environment, can let our students to try and make mistakes, and then start from a white to enter the middle of this field, and ultimately what he can do, maybe we are not particularly sure, but if we don’t try, we just conduct a cross-sectional survey, to do factor analysis… it’s definitely normal, but it’s hard for us to have our own hands-on experience, because we may look at other people a lot, but if you don’t put our own foot in it, you never know how these things are made, right?’*

*(N4)*

##### Human and Technology Optimization and Iteration Still Need to Be Considered

The vitality of AI products/systems lies in continuous optimization and iteration. In the process of the application, transformation, and promotion of nursing-related products and systems, the optimization and iteration between people and technology also needs to be considered.

*‘In fact, the real AI applied to the clinic, it actually needs a long run-in period, not as ideal as we think. Likewise, when we carried out it (AI-driven nursing research project) earlier in our hospital, in fact, there is a long period of ‘pain’… nurses in this system to do running-in (system optimization and iteration) is actually very painful.’*

*(N7)*

One of the solutions to shorten the run-in time is to actively participate, design, supervise, and improve.

*‘We design AI teaching system on the basis of high simulation, but also in the process of system research and development, we definitely need these senior clinical nurses to join, otherwise the developed system does not meet the clinical context, then the follow-up optimization problems will be more, why not empowered them earlier to participant in this process to improve the system, right?’*

*(N9)*

##### It Takes Time for Humans and Machines to Accept Each Other

In the application, transformation, and promotion of “human–technology–machine” interaction factors, we must consider that the mutual acceptance of human and machine takes time to precipitate; especially in the promotion of applications, the human–machine interaction burden has become an important consideration.

*‘The clinical nurses were very uncomfortable at first, they thought I was moving around with the computer like this (figures waved), and it was more convenient for me to write and draw by hand…’*

*(N7)*

*‘…I even think sometimes it [the systems developed] has a bit of a counter-effect, which is that care is not only for the patient, but also for the so-called intelligent system, which is now happening in the clinic.’*

*(N10)*

*‘Intelligent systems and products often require more time for clinical nurses to learn.’*

*(N9)*

“The mutual acceptance of human and machine” also includes the concept of the acceptance and operation acceptance of clinical practitioners.

*‘Some of the senior nurses adhere to a more traditional and rigid operational model, making it quite challenging for them to embrace new concepts and integrate innovative approaches into their established routines.’*

*(N7)*

This problem, in turn, is an opportunity, for example, to change the mindset of clinical practitioners through the continuous training of models.

*‘I believe it’s a reciprocal process; through continuous training and improvement, we gradually refine our skills, and it becomes more polished. Simultaneously, our mindset evolves, and we come to accept that it isn’t just a figment of our imagination. It’s not about moving from the countryside to a grand villa overnight, but rather recognizing that it provides the framework, and it’s up to us to construct it into a luxurious villa. It’s not about instant gratification, but about the journey of creation.’*

*(N7)*

The acceptance of operational habits should also be taken into account.

*‘I found that some senior practitioners, who are not frequent computer users, find it difficult and challenging for them to get started.’*

*(N12)*

##### Poor Resource Integration

The research and development of AI-driven nursing hinges on the “troika” of algorithms, computing power, and big data. However, currently, there is a significant deficiency in the integration of relevant resources within the country.

*‘There is a state of siloed, fragmented research, and if there is really going to be some kind of industry change, it needs to be at least at the level of, say, Dr. Watson, and probably a lot more integration of resources.’*

*(N6)*

*‘If you cannot achieve such a breakthrough: the network of cross-regional institutions, it is impossible to truly achieve big data, we are facing a substantial challenge…’*

*(N3)*

The promotion and application of products require additional support from external resources. For instance, the N11 project encountered audit-related challenges during its promotional phase.

*‘The products we have developed have been iterated generation after generation, and we do want to promote the application, unfortunately, a challenge emerged concerning the commercialization of the mobile phone system, necessitating audit compliance documents and legislative development by the principal organization. However, we are not incorporated as a legal entity, we’re just a couple of separate individuals involved in the research.’*

*(N11)*

#### 3.5.2. Financial Support and Continuous Investment

Research requires funding.

*‘All these things can be done, but the question is financial resource allocation.’*

*(N8)*

Further promotion also requires funds or market investment.

*‘Nursing presents significant opportunities for applied research, yet requires greater investment. Clinical diagnostic models are easily implemented, while our innovative small-scale models offer effective alternatives. The field’s multidisciplinary nature, integrating management with humanities and social sciences, presents unique challenges in health management development, which struggles to attract corporate investment due to its lower financial returns compared to the integrated medical-pharmaceutical sector.’*

*(N5)*

#### 3.5.3. The Controversy Behind the Intelligent Maturity Level

At present, the research and development of intelligent tools, products, and systems in the field of nursing is indeed in the ascendant, but the problem of intelligent “in-wisdom” is still prominent. Even the developed system still needs to improve the level of intelligent maturity.

*‘When we show (intelligent tool) on the spot, we must feel very good and dazzling, but when we use it in our daily work, we will still find that it has many technical constraints and system deficiencies., in fact, it does need to constantly upgrade the system, and there are still some problems with the designed prediction model.’*

*(N7)*

*‘Current mission robots operate effectively within their programmed corpus, delivering precise responses to trained queries. However, they face limitations when encountering questions outside their knowledge base (pause)…he is a bit silly, and he will tell you that I can’t answer and you need to find a human.’*

*(N8)*

*‘Currently, we’re only using several means of information technology related. We’re still far from fully leveraging AI, and the current intelligence is far from meeting the requirements of nurses…’*

*(N10)*

#### 3.5.4. Application Risk and Fault Tolerance

“Technology is a double-edged sword”, and the application of AI-related technologies in the field of nursing also needs to consider safety, risk, and accuracy.

*‘Are you willing to let these mechanical things to judge your life and death? This constitutes a critical issue requiring thorough examination, of course, AI can actually be used in many scenarios in health care, but it has risks, and which are not the same as other risks.’*

*(N6)*

*‘In fact, there’s a great deal that robot technology is capable of, but we have to evaluate the risk, human errors can be attributed to individuals, but machine errors often lead to irreversible consequences.’*

*(N2)*

*‘I think things in the medical field are different from a lot of other fields… if you use it to diagnose diseases, to do care programs, you ought to be 100% right, even if this model is 99.9% accurate, anyway, it might be 0.1% wrong…’*

*(N5)*

The accuracy of the developed models is closely related to the degree of application promotion.

*‘In specialized medical fields, current models fall short. They cannot determine precise statin dosages, assess minimal daily requirements, or guide treatment discontinuation in complex cases.’*

*(N5)*

*‘Some research papers appear highly impressive at first glance, particularly in fields like multimodal learning. However, they often lack practical applications. (Pause) In real-world business scenarios, such as clinical settings, where you need to integrate and analyze diverse data types-from metabolic profiles to protein data and beyond-is this truly feasible…’*

*(N10)*

*‘Is your model sufficiently accurate for commercial implementation? The market environment operates on a distinct paradigm from academic research, characterized by immediate user-driven selection processes based on practical efficacy, contrasting sharply with the more theoretical evaluation criteria prevalent in research contexts.’*

*(N5)*

## 4. Discussion

The evolution from the mechanical, electric, and information revolutions to the release of ChatGPT on 30 November 2022 marks the dawn of the Fourth Industrial Revolution. As a discipline closely tied to human health, nursing holds significant potential for growth and transformation through AI integration.

The findings from this study highlight AI’s strong prospects as a nursing support tool in assisting decision-making, assisting with writing nursing documents, assisting with nursing operations with exposure risks and physical exertion, and assisting with carrying out nursing activities, etc. The existing literature also underscores AI’s expanding role in nursing. A phenomenological study conducted by Badawy et al. among geriatric nurses revealed a general recognition of the potential of AI to enhance diagnostic accuracy, personalized care, continuous monitoring, and pattern recognition from clinical data. However, participants also expressed concerns regarding challenges such as workflow integration difficulties, cost-related barriers, resistance to change, data privacy issues, diminished humanistic care, and the lack of ethical guidelines. The nurses adopted a cautiously optimistic stance, emphasizing the importance of acknowledging real-world challenges, preserving humanistic values, and promoting a collaborative implementation approach in advancing AI applications [15]. João Ventura-Silva [23] identified the AI-mediated tools used in nursing work organization and summarized them into three tool models, namely monitoring and prediction, decision support, and interaction and communication technology. Howarth M [24] and Jauk S [25] conducted research on predictive models and auxiliary decision-making, and Koleck T et al. used natural language processing (NLP) to identify symptom information in nursing records [26]. Chang CY et al. [7] found, through experimental research, that generative AI based on ChatGPT has great potential in nursing education design courses, which can improve the shortcomings of traditional teaching methods, etc. An integrative review revealed that the potential applications of AI in mental health nursing are diverse, encompassing assessment, identification, prediction, optimization, and perception [27]. Al Khatib et al. reviewed the application of AI in nursing and its impact on the evolving role of nurses in patient care [28]. The findings from Hassanein’s integrative review indicate that the integration of AI into nursing practice has yielded significant clinical and operational benefits [29]. However, some studies have expressed concerns about the future use of AI-related technologies. Participants who had not directly interacted with AI systems and whose understanding was limited to literature or general knowledge tended to believe that AI and robotic nurses could potentially have a positive impact on healthcare delivery. Nevertheless, they also voiced apprehension about the inability of such technologies to replicate the “human touch,” particularly in domains that require empathy, emotional connection, and personalized care—areas where robotic capabilities remain notably limited [15,18]. With the growing body of research on AI-driven nursing, standardized guidelines are needed. To enhance the quality of AI-related research in nursing, studies must comprehensively address key elements, including research objectives, study contexts, methodologies, and ethical considerations [30].

The rise of AI-driven nursing technology is transforming traditional nursing research and practice, presenting both opportunities and challenges. Effectively integrating AI requires the active participation of key stakeholders throughout the entire process. It is essential to adopt a proactive approach that embraces intelligence and automation while ensuring a balanced and multidisciplinary response. This study supports the principle that “education and scientific research come first, a fully explore application scenarios, and deeply integration with multiple disciplines”, aligning with the existing literature [31,32,33]. Additionally, engaging end users in AI technology development is crucial [34], with co-engagement, co-design, co-supervision, and co-improvement serving as key strategies [35,36].

Addressing challenges in AI-driven nursing research requires multifaceted strategies. The corresponding strategies focus on the above four subthemes to make breakthroughs: optimizing human–technology–machine interactions in application and promotion, securing financial support and continuous investment, gradually improving the level of intelligent maturity, and carefully assessing application risks and fault tolerance.

Education plays a crucial role in optimizing human–technology–machine interactions in both the application and promotion of AI technologies. The target audience for such educational efforts is broad, encompassing nursing educators, clinical practitioners, and research developers—including students. Rony et al. emphasized the importance of enhancing nursing educators’ competencies to enable them to prepare future nurses who are well-equipped for AI-enabled clinical environments. This, in turn, fosters an effective alignment between education and the rapidly evolving landscape of healthcare practice [16].

Findings from a cross-sectional study further demonstrated that a positive attitude toward AI and active engagement in its use were significantly associated with increased intent to remain in the nursing workforce, while perceived barriers undermined job retention stability [11]. Therefore, it is essential to implement measures that enhance clinical nurses’ participation in the use of AI-related software and products. In addition, qualitative findings from Alenazi et al. suggest the necessity of integrating AI-related content into nursing curricula to adequately prepare students for the application of AI in future healthcare settings [37].

Ethical considerations remain critical. Nurses must be aware of potential risks and unintended consequences of AI technology, as highlighted by Ronquillo et al. [38] and echoed by participants in this study. Thus, AI should be applied rationally, prudently, and scientifically to maximize its benefits while minimizing risks in nursing research and practice.

### Limitations

In this study, two interviewees (N10 and N12) were interviewed via online video conferencing. It may not fully replace the sense of authenticity and intimacy of face-to-face communication. It may render the interaction between the interviewer and the interviewees rigid, thereby impeding the establishment of a profound trust relationship. Moreover, to a certain extent, it can also undermine the depth of the information shared by the interviewees.

Before interviewing, there was a certain level of familiarity between the researcher and the participants, which was intended to build trust and facilitate open, in-depth conversations. We believe this rapport encouraged participants to speak more freely and share richer insights. This point may also reflect potential influences on the data.

## 5. Conclusions

Through in-depth interviews with 12 nursing educators and clinical practitioners, this study explored perspectives on the integration of AI in nursing. Three key themes and eleven sub-themes were identified. While AI offers opportunities for nursing, its implementation requires an approach to ensure safe, effective, and ethical integration into clinical settings. To address the extracted challenges and accelerate the adoption of AI in nursing, we offer several actionable recommendations. For the academic field, establishing interdisciplinary research platforms and standardized evaluation frameworks for AI in nursing is crucial. Nursing education curricula should incorporate foundational AI literacy and ethical considerations by interdisciplinary education. For the healthcare service sector, hospitals and institutions should pilot AI-supported care models in selected clinical settings, while policymakers should allocate sustained funding to ensure equitable access, training, and implementation. Participants consistently emphasized the importance of an evidence-based, context-aware approach to AI adoption. Overall, the findings highlight the urgent need for comprehensive policy support, systematic educational framework in nursing education, and dedicated investment from both the academic field and the healthcare service sector to enable the responsible and sustainable integration of AI technologies into nursing science and practice.

## Figures and Tables

**Table 1:** Demographic data of the interviewees.

Number	Age	Gender	Education Level (Degree)	Occupation	Professional Title	Years of Experience in AI-Related Fields (Years)	Teaching Experience in Nursing Field (Years)
N1	45	Male	PhD	Medical informatization	Associate Professor	20	4
N2	35	Female	PhD	Teacher	Associate researcher	4	6
N3	33	Female	PhD	Teacher	Lecturer	5	3
N4	39	Female	PhD	Teacher	Associate Professor	3	13
N5	36	Male	PhD	Teacher	Associate Professor	3	5
N6	31	Female	PhD	Teacher	Lecturer	8	5
N7	34	Female	PhD	Nurse	In-charge Nurse Practitioner	4	5
N8	38	Female	PhD	Nurse	Associate Chief Nurse	4	15
N9	42	Female	PhD	Nurse	Chief Nurse	7	18
N10	60	Female	MD	Nurse	Chief Nurse	4	30
N11	35	Female	PhD	Teacher	Associate Professor	7	8
N12	38	Male	PhD	Nurse	Associate Chief Nurse	5	10

**Table 2:** The process of refining the theme.

Selected Participant Quotes	Sub-Themes	Major Themes
1-1-1 *‘We can use clinical data to quickly make intelligent recommendations based on certain rules’ (N11 & N12)*	1-1 Aid in decision-making	1. The potential of multi-perspective development of artificial intelligence-empowered nursing science and practice
1-2-1 *‘A nurse would have to record a lot of things every day, if there were a system to record intelligently…’ (N1 & N8)*	1-2 Assisting with writing nursing documents	
1-3-1 *‘We implement these projects, I think it will reduce some of the repetitive labor of medical staff’ (N1 & N 7& N11 & N12)*	1-3 Help in care practices with high exposure risks and heavy physical exertion	
1-4-1 *‘The nursing robot can feeding the patients’ (N10), ‘In the aging society, the use of nursing robots to care for the elderly will have a huge demand, now young people are very busy, many old people have nobody to take care of, and the caregiver is not so reliable, compared with nurses…’ (N1)*	1-4 Support the development of nursing activities	
2-1-1 *‘Only with prior scientific research can we further implement it into practice.’‘ In promoting the application of AI technology into nursing science, talent training is the key’ (N6 & N10)*	2-1 Education and scientific research come first	2. Multi-dimensional response to the wave of intelligent nursing research and practice
2-2-1 *‘Currently, the students should cultivate the ability to figure out that which situation is suitable to integrate with the artificial intelligence techniques’ (N2 & N3 & N6)*	2-2 Fully explore the application scenarios	
2-3-1 *‘If there is a long-term and stable cooperative relationship with the computer information technology team, the cooperation process will be very efficient. For example, I put forward the idea of the product I want, including the path I expect, and then the computer information technology team proceeds to further implement it’ (N6 & N11)*	2-3 Deeply interdisciplinary integration	
*3-1-1 ‘Perhaps nursing experts, professors and pioneers in the nursing field also need to advocate for a more liberal and relaxed environment, enabling everyone to make more attempts.’ (N4 & N5) ‘We experienced an extremely tough and long adjustment period to promote the projects’ (N7 & N10 & N11)*	3-1 Interaction factors of “human–technology–machine” for application, transformation and promotion	3. Obstacles to intelligent nursing research and practice
*3-2-1 ‘Since the outbreak of the Covid19 pandemic, there has been a certain tightness in funds. As a result, the development of intelligence in our hospital is evidently not as vigorous as it was in previous years.’ (N8)*	3-2 Financial support and continuous investment	
3-3-1 *‘In fact, I think that in a sense, the gap between the so-called artificial intelligence nowadays and true artificial intelligence is still quite vast.’ (N10 & N5)*	3-3 The controversy behind the intelligent maturity level	
3-4-1 *‘Would you be willing to let these mechanical things determine your life and death, as well as your health?’ (N5 & N6)*	3-4 Application risk and fault tolerance	

## Data Availability

Due to confidentiality and privacy protections, the raw data supporting this study cannot be made publicly available. However, anonymized datasets may be made available from the corresponding author upon reasonable and scientifically justified request, subject to ethical review.

## Public Involvement Statement

No public involvement in any aspect of this research.

## Guidelines and Standards Statement

Reporting adheres to the consolidated criteria for reporting qualitative research (COREQ) checklist. A complete list of reporting guidelines can be accessed via the equator network: https://www.equator-network.org/ (accessed on 12 June 2025).

## Appendix A. Interview Outline

1. How did you initially learn about artificial intelligence (AI)?
2. How would you describe your understanding of artificial intelligence (AI) in nursing?
3. What do you think of the role of artificial intelligence in nursing science?
4. Can you share your personal experiences of encountering or interacting nursing projects/practices with AI technology?
5. Based on your experiences, what do you think are the potential benefits and difficulties that come with incorporating AI into nursing projects/practices?
6. What do you learn from your project that incorporates AI technology? Which particular aspect left the deepest impression on you?
7. What do you think about the cooperation between AI technology and human nurses?
8. How can nursing students/beginner best prepare to adapt to these changes?
9. Contemplate your experience and insights, for nursing education, how to develop a plan for AI education and training to improve the understanding and application of AI in nursing students?
10. Do you have any suggestions or strategies to facilitate a smooth transition to an AI-empowered nursing environment in China?
